# Salvianolic Acid B Reducing Portal Hypertension Depends on Macrophages in Isolated Portal Perfused Rat Livers with Chronic Hepatitis

**DOI:** 10.1155/2012/786365

**Published:** 2012-10-16

**Authors:** Xin Zhao, Hongmei Jia, Shijun Yang, Yuetao Liu, Bo Deng, Xueyan Xu, Tao Zhang, Hang Zhou, Chengzhe Zu, He Yin, Ting Li, Yijun Song, Yueqi Wang, Pengtao Li, Zhongmei Zou, Dayong Cai

**Affiliations:** ^1^Institute of Medicinal Plant Development, Chinese Academy of Medical Sciences, Peking Union Medical College, Beijing 100193, China; ^2^School of Basic Medicine, Beijing University of Chinese Medicine, Beijing 100029, China

## Abstract

This study is aimed to investigate the effects of Sal B on portal hypertension (PH). PH with chronic hepatitis was induced by carbon tetrachloride (CCl_4_) in rats. The model was confirmed with elevated portal pressures and increased serum CD163 levels. The inducible nitric oxide synthase (iNOS) or heme oxygenase-1 (HO-1) in portal triads was assessed. The isolated portal perfused rat liver (IPPRL) was performed at *d*_0_, *d*_28_, *d*_56_ , and *d*_84_ in the progression of chronic hepatitis. After constricting with phenylephrine, the portal veins were relaxed with Sal B. The EC_50_ of Sal B for relaxing portal veins was −2.04 × 10^−9^, 7.28 × 10^−11^, 1.52 × 10^−11^, and 8.44 × 10^−11^ mol/L at *d*_0_, *d*_28_, *d*_56_, and *d*_84_, respectively. More macrophages infiltrated in portal triads and expressed more iNOS or HO-1 as PH advanced. The areas under the curve (AUCs) of Sal B for reducing PH were positively correlated with the levels of iNOS or HO-1 in portal triads, and so did with serum CD163 levels. Sal B reduces PH in IPPRL with chronic hepatitis, via promoting portal relaxation due to macrophage-originated NO or CO in portal triads, partly at least.

## 1. Introduction

Portal hypertension (PH) is a common complication in the patients with advanced chronic hepatitis [1]. The increased hepatic vascular resistance and portal hyperemia are involved in the reversible pathogenesis as the potent therapy targets [2]. 

Salvianolic acid B (Sal B) is a molecule from the root of *Salvia miltiorrhiza* (Danshen), which is a traditional Chinese medicine widely used for cardiovascular diseases [3]. Sal B is effective for liver fibrosis and PH in patients [4] or animals [5]. In the endothelin_−1_-induced PH rats, Sal B could inhibit the constriction of hepatic stellate cells [5]. However, our previous study indicated that Sal B constrict portal veins of the isolated portal perfused rat livers (IPPRLs) at physiological status [6]. The underlying mechanisms of Sal B for PH remain unclear.

It is reported that nitric oxide (NO) and carbon monoxide (CO) play key roles in the pathogenesis of PH [7]. Both signal molecules directly relax portal veins through upregulation of cGMP via guanylate cyclase [8]. NO from endothelial NO synthase (eNOS) aggravates PH through systemic hyperemia [9], and inducible NO synthase (iNOS) exacerbates PH by producing peroxynitrite (ONOO^−^) [10]. It has been reported that the reduced NO bioavailability is involved in the increased hepatic vascular resistance [11]. There is an increase of superoxide release by NADPH oxidase in liver with chronic hepatitis [12] and an overproduction of iNOS from macrophages [10]. The iNOS-derived NO reacts with superoxide, leading to ONOO^−^ formation, with a decrease in NO bioavailability [10]. Heme oxygenase-1(HO-1) is a rate-limiting enzyme catalyzing heme to CO, iron, and biliverdin. Biliverdin is then converted to bilirubin, which acts as a highly effective antioxidant and free radical scavenger against oxidation [13]. HO-1 also showed hepatoprotection against ischemia-reperfusion injury, endotoxemia, hyperoxia-induced hepatic injury, and immune-mediated apoptotic liver damage [14]. Furthermore, HO-1/CO activation downregulates the inflammatory response by blocking the formation of ONOO^−^ from iNOS [13]. While the ONOO^−^ induces HO-1 protein expression but mediating its inactivation [15]. 

Sal B has an effect on [3] production of NO or CO from activated macrophages [16] under inflammatory cytokines [17]. In addition, Sal B could protect endothelia from the oxidation by blocking PI3K/Akt signal pathway [18]. Therefore, Sal B was proposed to rescue NO bioavailability or to maintain CO potency from the macrophage at portal triads in advanced chronic hepatitis.

The purpose of present study is to investigate the effects of Sal B on PH in IPPRL with chronic hepatitis and to analyze further the NO or/and CO signals through the relationship between the Sal B potency and the existed iNOS or HO-1 from the macrophages in portal triads.

## 2. Materials and Methods

### 2.1. Reagents

Carbon tetrachloride (CCl_4_), olive oil, and heparin sodium were purchased from Sinopharm Chemical Reagent Company. Acetylcholine chloride and phenylephrine hydrochloride were obtained from Sigma (USA). Salvianolic acid B (purity >99%) was purchased from Shanghai Institute of Materia Medica, Chinese Academy of Sciences.

### 2.2. Animals

Thirty two six-week-old male Wistar rats (180–200 g) were purchased from Animal Centre of the Chinese Academy of Medical Sciences. All rats were kept under a 12 h/12 h light/dark cycle, temperature (25.0 ± 0.2°C), and humidity (45 ± 2%) controlled SPF environment. The rats were fed standard rodent pellets and allowed free access to filtered water. All experiment procedures were performed in accordance with the Guidelines of Animal Experiments from the Committee of Medical Ethics, National Health Department of China.

### 2.3. Induction of Portal Hypertension with Chronic Hepatitis

**Figure 1:**
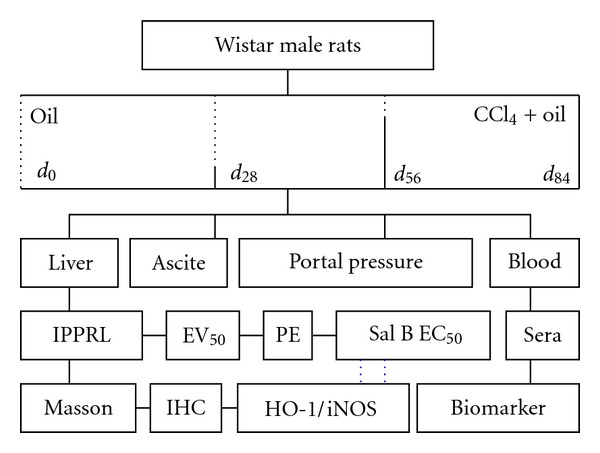
Experimental design about Sal B relaxing rat portal hypertension. IPPRL, isolated portal perfuse rat liver; EV_50_, median effective velocity; PE, phenylephrine; EC_50_, median effective concentration; Sal B, salvianolic acid B; Masson, Masson's trichrome; HO-1, heme oxygenase-1; iNOS, inducible nitric oxide synthase; IHC, immunohistochemistry; AUC, area under the curve.

PH with chronic hepatitis was induced by CCl_4_ in rat as described previously (Figure 1) [19]. Rats were injected subcutaneously with a mixture of 40% (v/v) CCl_4_ in olive oil (3 mL/kg) two times a week for 0, 28, 56, and 84 days, respectively [20, 21]; olive oil was the vehicle for age-matched control. Eighty four hours after the last CCl_4_ injection, rats were anesthetized with a subcutaneous injection of sodium pentobarbital (50 mg/kg). A midline incision was made to open abdominal cavity, and ascitic samples were collected and quantified as described preciously [22]. The exuded liquid ratios were calculated as *exuded liquid weight/body weight ×100*. The portal pressure in vivo was recorded. The blood sample was collected for analyzing the serum alanine aminotransferase (ALT), aspartate aminotransferase (AST), alkaline phosphatase (ALP), and albumin (Alb) levels by biochemistry and CD163 levels by immunoassay. Then the hepatic artery, portal vein, and hepatic vein were canalized [20, 21]. The liver, spleen, and kidneys were harvested, and the organ indexes were calculated as *organ weight/body weight ×100*.

### 2.4. CD163 Immunoassay

The blood samples were centrifuged at 1200 g for 10 min at 4°C, and serum was stored at −80°C until the assays. Serum CD163 levels was measured using enzyme-linked immunosorbent assay kits (R&D Systems, Wiesbaden, Germany) following the manufacturer's guidelines. 

### 2.5. Histological and Morphometry

#### 2.5.1. Histological Quantification

Formalin-fixed, paraffin-embedded liver sections were cut at a 6 *μ*m thickness and then stained with Masson's trichrome (Masson) [19]. Images were obtained using NanoZoomer Digital Pathology system (Hamamatsu, Japan). The collagen density was quantified using Image ProPlus analysis system 7.0.1 (no. 41N70000-60555, Media Cybernetics, USA) at 100 × magnification. The data were expressed as the one-ten thousandth of collagen (the ratio of collagen area per total analyzed field area ×1000%). Values are expressed as the average of ten fields taken from each section.

#### 2.5.2. Immunohistochemistry for Localization and Quantification of iNOS and HO-1

For immunohistochemical analysis, sections were incubated with rabbit polyclonal antibody against iNOS (1 : 500 dilution, sc-8310, Santa Cruz Biotechnology) or HO-1 (1 : 200 dilution, sc-10789, Santa Cruz Biotechnology). Staining was visualized using avidin-biotin peroxidase immunostaining kit with diaminobenzidine (Boster, Wuhan, China). The mean optical density (OD), positive staining area (A_P_), and observed area (A_T_) were determined with Image ProPlus 7.0.1 at 400 × magnifications. The levels of iNOS and HO-1 were calculated by the formula [OD × (A_P_/A_T_)^3/2^]. The average of ten random fields generated a single data for statistic analysis [19].

### 2.6. Effect of Salvianolic Acid B on Isolated Portal Perfused Rat Livers with Chronic Hepatitis

The isolated portal perfusion system was performed with controlled velocity as described previously [20, 21]. At *d*_0_, *d*_28_, *d*_56_, and *d*_84_, the perfuse velocity was chosen 3935.50, 4720.63, 4753.35, or 5164.16 (*μ*L/min), respectively [20, 21]. Phenylephrine hydrochloride was determined as 1.69 × 10^−10^, 2.64 × 10^−10^, 5.82 × 10^−10^, and 8.24 × 10^−10^ mol/L, respectively, to constrict portal veins [21]. After the phenylephrine constriction, Sal B (10^−13^–10^−7^ mol/L) was added into the recirculating perfusate. Dose-response curves were obtained from the Sal B concentrations and the changed percentage of the perfused pressure from the baseline of phenylephrine constriction.

### 2.7. Statistical Analysis

All data are expressed as mean ± S.E.M. Comparisons between groups were performed using Student's *t*-test or Mann-Whitney. Significant differences were established at the 0.05 level. The equation, the EC_50_ with its 95% confidence intervals of Sal B, and the area under the curve (AUC) of Sal B were analyzed using GraphPad Prism 4 (GraphPad Software). The EC_50_ of Sal B (*y*) was regressed with the durations (0, 28, 56, and 84 days) and serum CD163 levels (*x*) in the progression of chronic hepatitis, and the AUC of Sal B (*y*) was regressed with the amounts of existed iNOS or HO-1 from immunohistochemical staining and the serum CD163 levels (*x*).

## 3. Results

### 3.1. General Characterization of Rats

**Figure 2:**
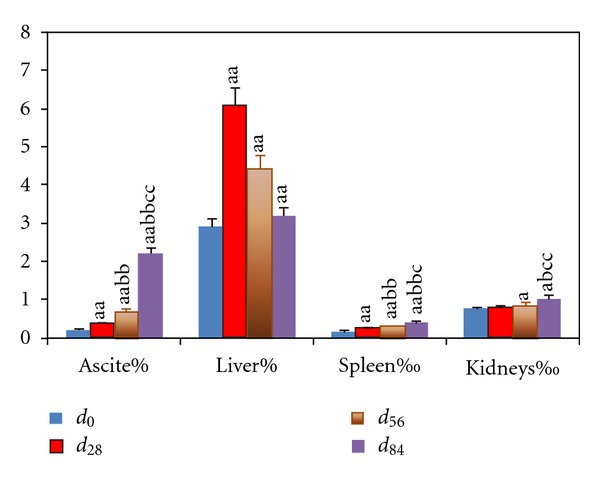
Ascite levels and organ indexes in portal hypertensive rats with chronic hepatitis. Data represent mean ± S.E.M. (*n*_*i*_ = 8). ^a^ *P* < 0.05, ^aa^ *P* < 0.01 compared with rats at *d*_0_; ^b^ *P* < 0.05 and ^bb^ *P* < 0.01 compared with rats at *d*_28_; ^c^ *P* < 0.05 and ^cc^ *P* < 0.01 compared with rats at *d*_56_.

**Figure 3:**
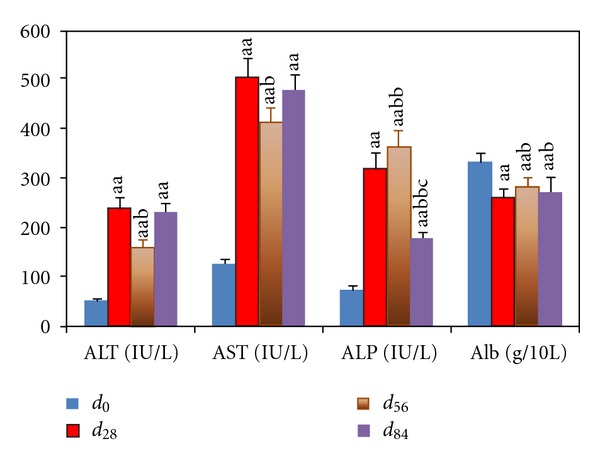
Serum ALT, AST, ALP, and Alb levels in portal hypertensive rats with chronic hepatitis. Data represent mean ± S.E.M. (*n*_*i*_ = 8). ^a^ *P* < 0.05, ^aa^ *P* < 0.01 compared with rats at *d*_0_; ^b^ *P* < 0.05 and ^bb^ *P* < 0.01 compared with rats at *d*_28_; ^c^ *P* < 0.05 and ^cc^ *P* < 0.01 compared with rats at *d*_56_.

The model of rat PH was confirmed by ascite levels, organ index, and serum biomarker levels (Figure 2). The exuded liquid ratios significantly elevated from *d*_0_ to *d*_84_ (*P* < 0.01) as the progression of chronic hepatitis (Figure 2). Hepatic indexes were the lowest at *d*_0_, the highest at *d*_28_, and reduced at *d*_56_ and *d*_84_ gradually (Figure 2). The splenic or renal indexes increased gradually from *d*_0_ to *d*_84_ (Figure 2). Serum ALT and AST levels increased from *d*_0_ to *d*_28_, then relived at *d*_56_ and *d*_84_. Serum ALP levels increased from *d*_0_ to *d*_56_, then relived at *d*_84_. Serum Alb levels decreased from *d*_0_ to *d*_28_, then relived at *d*_56_ and *d*_84_ (Figure 3).

### 3.2. Portal Pressure and Serum CD163 Levels

#### 3.2.1. Portal Pressure

**Figure 4:**
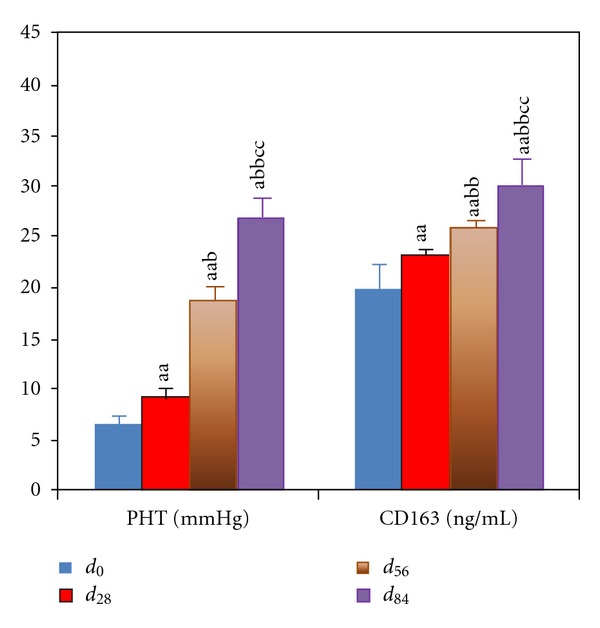
Changes of portal pressure and serum CD163 levels in portal hypertensive rats with chronic hepatitis. Data represent mean ± S.E.M. (*n*_*i*_ = 8). ^a^ *P* < 0.05, ^aa^ *P* < 0.01 compared with rats at *d*_0_; ^b^ *P* < 0.05 and ^bb^ *P* < 0.01 compared with rats at *d*_28_; ^c^ *P* < 0.05 and ^cc^ *P* < 0.01 compared with rats at *d*_56_.

The portal pressure in vivo significantly increased from *d*_0_ to *d*_84_ (*P* < 0.01) as the procession of chronic hepatitis (Figure 4).

#### 3.2.2. Serum CD163 Levels

CD163 is a biomarker of the activated macrophages in PH. The serum CD163 levels were increased gradually from *d*_0_ to *d*_84_ (*P* < 0.01) as the procession of chronic hepatitis (Figure 4).

### 3.3. Pathological Changes and Morphometry

#### 3.3.1. Pathological Changes

**Figure 5:**
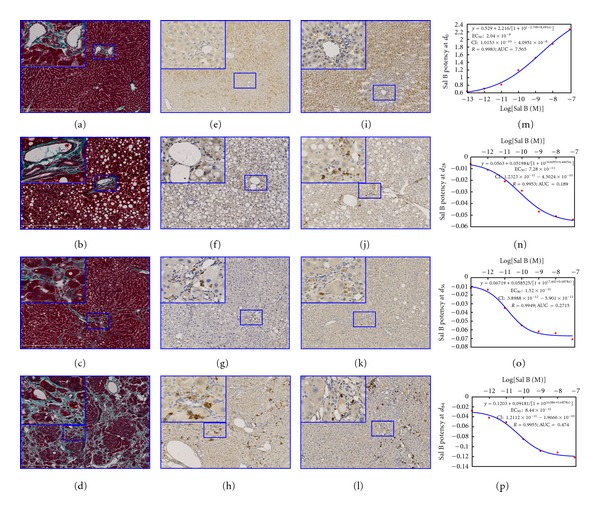
**: **Salvianolic acid B reducing portal hypertension in IPPRL with chronic hepatitis. (1) Masson staining was performed to evaluate collagen deposition (100×). (a) Liver with normal structure in normal rats at *d*_0_. (b) Hepatic degeneration in the portal hypertensive rats at *d*_28_. (c) Hepatic fibrosis in the portal hypertensive rats at *d*_56_. (d) Hepatic cirrhosis in the portal hypertensive rats at *d*_84_. The inserted micrographs in the upper left corner were the portal triad (Masson × 630) from the original ones (black rectangle). (2) Existence of iNOS was detected by immunohistochemistry staining (400×). (e) iNOS was located at the hepatocyte at *d*_0_. (f) iNOS was located at stellates in the lobules at *d*_28_. (g) iNOS was located at stellates and macrophages at *d*_56_. (h) iNOS was located at macrophages out lobules at *d*_84_. The inserted micrographs in the upper left corner were the portal triad (630×) from the original ones (Black rectangle). (3) Existence of HO-1 was detected by immunohistochemistry staining (400×). (i) HO-1 was located at the hepatocytes only at *d*_0_. (j) HO-1 was located at the macrophages in portal triads with less at the hepatocytes at *d*_28_ than that at *d*_0_. (k) HO-1 was located at the macrophages out lobules with lest at the hepatocytes at *d*_56_ among the durations of chronic hepatitis. (l) HO-1 was located at the macrophages in portal triads only at *d*_84_. The inserted micrographs in the upper left corner were the portal triad or its partners (630×) from the original ones (black rectangle). (4) (m) Sal B increased the portal pressure in the IPPRL at *d*_0_. (n) Sal B decreased the portal pressure in the IPPRL at *d*_28_. (o) Sal B decreased the portal pressure in the IPPRL at *d*_56_. (p) Sal B decreased the portal pressure in the IPPRL at *d*_84_.

The hepatic pathological changes induced by CCl_4_ were evaluated by Masson-stained sections. At *d*_0_, the liver showed normal hepatic architecture, and the collagen only normally distributed at the portal areas and around vessels (Figure 5(a)). At *d*_28_, the hepatic fatty degenerations and cellular swellings were obviously observed, and the hepatic sinusoid was severely narrowed without obvious collagen (Figure 5(b)). At *d*_56_, the hepatic fibrosis was observed and the collagen increased and mainly deposited in lobules. The relived enlarged hepatic cords led to the hepatic sinusoid widen obviously. Some deposited collagen in interlobular had extended into and separated lobules incompletely, thus the directions of blood flow were not changed in hepatic sinusoid (Figure 5(c)). At *d*_84_, the hepatic cirrhosis was evident. The lobules were completely destructed by deposited collagen and the formation of pseudolobules was observed, so the directions of blood flow were completely changed in hepatic sinusoid (Figure 5(d)).

#### 3.3.2. Collagen Ratio

**Figure 6:**
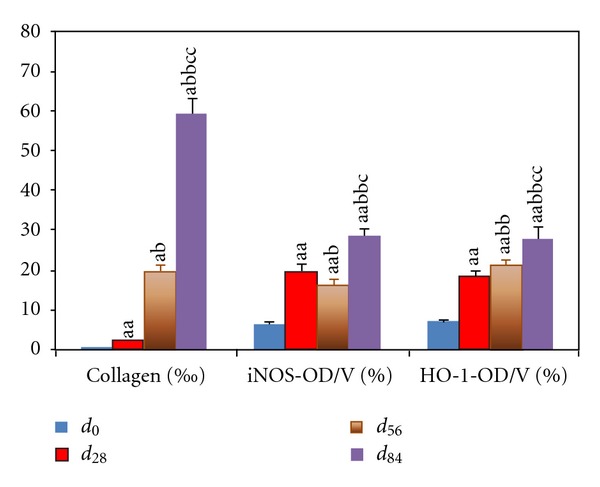
Collagen ratio and the levels of iNOS and HO-1 in portal triads. Data represent mean ± S.E.M. (*n*_*i*_ = 8). ^a^ *P* < 0.05, ^aa^ *P* < 0.01 compared with rats at *d*_0_; ^b^ *P* < 0.05 and ^bb^ *P* < 0.01 compared with rats at *d*_28_; ^c^ *P* < 0.05 and ^cc^ *P* < 0.01 compared with rats at *d*_56_.

Quantification of Masson staining by morphometry analysis showed that collagen ratios were increased along with the progression of chronic hepatitis (Figure 6). 

### 3.4. Localization and Quantification of Synthases

#### 3.4.1. iNOS Cellular Localization

The iNOS positive cells were the hepatocytes and scattered stellates in the lobules of the normal rats at *d*_0_ (Figure 5(e)). The iNOS expression was reduced in the scattered hepatocytes and mainly observed in stellates cells in the lobules of the rats with chronic hepatitis at *d*_28_ (Figure 5(f)). The expression of iNOS was completely quenched in the hepatocytes; the positive cells were the macrophages in the portal triads and the stellates in the lobules at *d*_56_ (Figure 5(g)). The main thick positive cells were the macrophages in the fibrous interval pseudolobules around vessels and the stellates with thin granules in the lobules at *d*_84_ (Figure 5(h)).

#### 3.4.2. iNOS Quantification

The iNOS-IHC OD per volume (Figure 6) in the portal triads of the rats with chronic hepatitis was significantly increased at *d*_28_ (2-fold), *d*_56_ (1.5-fold), and *d*_84_ (3-fold) compared with that at *d*_0_, respectively, (*P* < 0.01); these were decreased at *d*_56_ and increased at *d*_84_ compared with that at *d*_28_, respectively (*P* < 0.01); so did increased that at*d*_84_ compared with that at *d*_56_ (*P* < 0.01).

#### 3.4.3. HO-1 Cellular Localization

The main HO-1 positive cells were the hepatocytes near the central vein and the scattered stellates in the lobules, but the hepatocytes next to portal triads were absolutely negative in the normal rats at *d*_0_ (Figure 5(i)). Besides of the thinner granules in the hepatocytes, the main positive staining cells were the stellates in the lobules at *d*_28_ (Figure 5(j)). The thin granules have completely disappeared in the hepatocytes, while the positive cells were the macrophages in portal triads and the stellates in the lobules at *d*_56_ (Figure 5(k)). The main thick positive cells were the macrophages in the fibrous intervals out pseudolobules at *d*_84_ (Figure 5(l)).

#### 3.4.4. HO-1 Cellular Quantification

The total HO-1-IHC OD per volume (Figure 6) in the rats with chronic hepatitis was significantly increased at *d*_28_ (1.6-fold), *d*_56_ (2-fold), and *d*_84_ (3-fold) compared with that at *d*_0_, respectively (*P* < 0.01); these at *d*_56_ and *d*_84_ significantly increased compared with that at *d*_28_, respectively (*P* < 0.01); so did that at *d*_84_ compared with that at *d*_56_ (*P* < 0.01).

### 3.5. Salvianolic Acid B Reducing PH

#### 3.5.1. Dose-Effective Relation for Relaxing Portal Vein

At *d*_0_, Sal B constricted portal veins of normal rats (Figure 5(m)), the equation was *y* = 0.5290 + 2.2160/[1 + 10^(−2.7690+8.691*x*)^] (*R* = 0.9983, *P* < 0.01); the EC_50_ with its 95% confidence intervals was 2.04 × 10^−9^ (1.02 × 10^−10^–4.10 × 10^−8^) mol/L. At *d*_28_ (Figure 5(n)), *d*_56_ (Figure 5(o)), and *d*_84_ (Figure 5(p)) of the progression in the rats with chronic hepatitis, Sal B relaxed portal veins, the equations were *y* = −0.0563 + 0.0520/[1 + 10^(4.6695+0.4605*x*)^] (*R* = 0.9953, *P* < 0.01), *y* = −0.0672 + 0.0585/[1 + 10^(7.4420+0.6878*x*)^] (*R* = 0.9949, *P* < 0.01), and *y* = −0.1203 + 0.0918/[1 + 10^(6.0860+0.5903*x*)^] (*R* = 0.9955, *P* < 0.01), respectively; the EC_50_ with their 95% confidence intervals were 7.28 × 10^−11^(1.23 × 10^−11^–4.30 × 10^−10^) mol/L, 1.52 × 10^−11^(3.90 × 10^−12^–5.90 × 10^−11^) mol/L, and 8.44 × 10^−11^(1.21 × 10^−11^–1.97 × 10^−10^) mol/L, respectively.

#### 3.5.2. Time-Effective Relation with Pathological Progression

The liner regressive equation was *y* = 2.2170*x* − 140 (*R* = 0.7861, *P* < 0.05) from the EC_50_ (*y* × 10^−11^ mol/L) of Sal B to the durations (*x* = *d* × 24 + 11.47 (hours); *d* = 0, 28, 56, and 84 days) of chronic hepatitis progression. So did the equation was *y* = 201.9300*x* − 3993 (*R* = 0.9982, *P* < 0.01) from CD163 levels to the durations.

#### 3.5.3. Salvianolic Acid B-AUCs Correlated with Existed iNOS

The liner regressive equation was *y* = 0.3587*x* − 8.0364 (*R* = 0.83391, *P* < 0.05) from the AUCs of Sal B to the iNOS-OD/V (%) in portal triads at *d*_0_, *d*_28_, *d*_56_, and *d*_84_ in the progression of CCl_4_-induced chronic hepatitis.

#### 3.5.4. Salvianolic Acid B-AUCs Correlated with Existed HO-1

The liner regressive equation was *y* = 0.4120*x* − 9.3727 (*R* = 0.9062, *P* < 0.05) from the AUCs of Sal B to the HO-1-OD/V (%) in portal triads at *d*_0_, *d*_28_, *d*_56_, and *d*_84_ in the progression of CCl_4_-induced chronic hepatitis. 

#### 3.5.5. Salvianolic Acid B-AUCs Correlated with Serum CD163 Levels

The liner regressive equation was *y* = 0.8531*x* + 26.2360 (*R* = 0.7838, *P* > 0.05) from the AUCs of Sal B to the serum CD163 levels at *d*_0_, *d*_28_, *d*_56_, and *d*_84_ in the progression of CCl_4_-induced chronic hepatitis. It was *y* = 22.8210*x* + 19.3530 (*R* = 0.9889, *P* < 0.01) from the AUCs to the serum CD163 levels at *d*_28_, *d*_56_, and *d*_84_ in the progression of CCl_4_-induced chronic hepatitis.

## 4. Discussion 

It was demonstrated in the present study that Sal B relaxed portal veins in IPPRLs of CCl_4_-induced chronic hepatitis. Its mechanisms are related to the inhibition of oxidative stress from macrophages and the increase of NO bioavailability or CO potency in portal triads. Sal B is the most active antioxidant extracted from Danshen and has obvious effects for liver fibrosis, chronic hepatitis, or PH in clinic [4].The mechanisms responsible for the protective effects of Sal B in PH remain unclear.

It has been reported that the portal resistance is mainly located at the terminal portal venules (TPV) in portal triads [23]. The activated macrophages release vasoactive substances concomitantly and increase the perfusion resistance [24]. Accordingly, we have previously demonstrated that the macrophages out lobules express more iNOS, produce more NO, and generate ONOO^−^ to further reduce NO bioavailability and aggregate PH [22]. The HO-1/CO activation decreases iNOS expression, enhances antioxidative effect, and upregulates extracellular superoxide dismutase (ecSOD) [13]. The local ecSOD could scavenge superoxide and block ONOO^−^ generation [11]. Therefore, macrophage-derived NO or CO in portal triads was considered as the most effective target. Sal B, a molecule from medical plants [3] for PH [4–6, 20, 21, 25], has benefits to elevate NO bioavailability and to maintain CO potency [17], and to inhibit oxidation [18], especially in macrophages [16]. We hypothesized that Sal B relaxes portal veins through anti-oxidative effects on NO or CO potency.

In present study, PH model in rat was replicated by CCl_4_-induced chronic hepatitis and was confirmed with increased portal pressure and pathological changes, such as the peritoneal exudation, enlarged hepatic indexes, serum hepatic biomarker levels, collagen deposition, and pseudolobule formation [1, 2]. The serum CD163 levels increased as the procession of chronic hepatitis, indicating the activation of macrophages, which was consistent with the PH patients [24]. We found Sal B reduced PH as a candidate from a medical plant for PH patients. Sal B increased the portal pressure of the IPPRLs at physiological status and reduced the PH of the IPPRLs at chronic hepatitis status in this study. EC_50_ of Sal B relaxation was positively correlated with the duration of CCl_4_-induced chronic hepatitis, indicating the action of Sal B which was pathological dependent. Our results demonstrated that increased iNOS or HO-1 levels in the macrophages infiltrated in portal triads are involved in the mechanism of Sal B relaxation. The existed levels of iNOS or HO-1 in lobules disappeared gradually, these in portal triads strengthened continuously along with the progression of CCl_4_-induced chronic hepatitis, especially in the infiltrated macrophages. We also reported here that iNOS and HO-1 levels in portal triads are correlated positively with the AUCs of Sal B for reducing PH.

**Figure 7:**
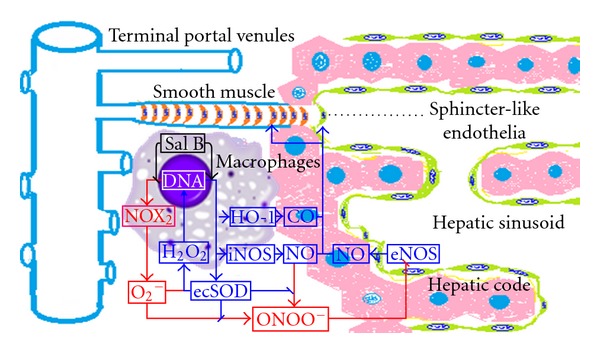
Salvianolic acid B indirectly reduced portal hypertension via NO/CO potency. In portal triads, the smooth muscle cells in terminal portal venules and the sphincter-like endothelia at hepatic sinusoid inlets were relaxed by both of the nitric oxide (NO) from inducible NO synthase (iNOS) or endothelial NO synthase (eNOS) and the carbon monoxide (CO) from heme oxygenase-1 (HO-1). The resistance of intrahepatic portal vein was mainly originated from both cells, since the expanding portal vein in portal triads indicated its backward location, and the narrowed hepatic sinusoids between hepatic cords did its forward location. It was the infiltrated macrophages that make salvianolic acid B (Sal B) effect switching, from its constricting in physiological status into its relaxing terminal portal venules in chronic hepatitis. The mechanisms of Sal B might be exploited from its elevating NO bioavailability and CO potency. (1) Sal B limited NADPH oxidase (NOX_2_) expression, decreased the superoxide (O_2_^−^), blocked peroxynitrite (ONOO^−^) generation from NO, and inhibited eNOS nitration and inactivation. (2) Sal B promoted iNOS/HO-1 expression and increased NO/CO potency to relax the smooth muscle cells and the sphincter-like endothelia. (3) Sal B might regulate upward the extracellular superoxide dismutase (ecSOD), promoted H_2_O_2_ formation from O_2_^−^, blocked ONOO^−^ generation from NO, then H_2_O_2_ enhanced iNOS, HO-1 and ecSOD expression itself. It consisted of a benign cycle (blue lines) against the vicious cycle (red lines).

The IPPRL was used in this study to evaluate the effect of Sal B on PH. The hepatic artery was ligated to ensure that the portal resistance originated mainly from the smooth muscle cells in terminal portal venule (TPV) and the sphincter-like endothelia at hepatic sinusoid inlets [23]. In PH rodents, the TPVs were the major resistance in portal microcirculation without enough collateral (like pre-TPV) or sinusoidal (post-TPV) networks to compensate a blood pressure increase [26]. Furthermore, the infiltrated activated macrophages in portal triads were next to TPVs in the rats with oxidative chronic hepatitis (Figure 7 inserted micrographs). Sal B relived endothelin-induced elevated portal pressure in physiological rats [27] or mice [28]; these did not agree with the data in this research that Sal B increased further phenylephrine-induced elevated portal pressure in the rats without chronic inflammation [6, 20, 21]. It suggested that the macrophages infiltrated in portal triads being the indirect cellular targets of Sal B to reduce PH in the rats with chronic hepatitis. There were at least four possible pathways for Sal B decreasing PH from oxidative chronic hepatitis (Figure 7). (1) NO signal: Sal B inhibited oxidative stress of activated macrophages [17], blocked ONOO^−^ generation [3], rescued iNOS activity from the inactivation by nitrate modification [10], and consequently increased local NO level to relax the TPV. Especially the AUC of Sal B for reducing PH correlated positively with the existed level of iNOS from the macrophages [8–11, 23]. Sal B relaxed indirectly portal vein via restoring NO bioavailability [29]. (2) CO signal: Sal B increased the expression of HO-1 from activated macrophages [16] and elevated local CO level to dilate TPV [8, 17]. Especially the AUC of Sal B for reducing PH correlated positively with the existed level of HO-1 from the macrophages in the portal triads. Meanwhile, HO-1-derived bilirubin directly inhibits NADPH oxidase and increases ecSOD and then decreases superoxide production and ONOO^−^ formation [13]. (3) EcSOD protection: Sal B might indirectly upregulate ecSOD expression, which converts superoxide to hydrogen peroxide and blocks ONOO^−^ generation from NO [11, 30]. Then the hydrogen peroxide could enhance iNOS, HO-1, and ecSOD expression itself to against the vicious cycle in PH. (4) Calcium signal: being considered as a cardiovascular protective agent [31], Sal B acted on TPV endothelia and smooth muscles. On human endothelia, Sal B activated transcription factor 4 or 6, consequently regulated upwards glucose-regulated protein 78, to protect the cellular damage from oxidative stress [30]; Sal B suppressed JAK/STAT1 activation in endothelia to relive vessel inflammation [32]. On human vascular smooth muscle, Sal B limited calcium channel to decrease Ca^2+^ influx [33]. It's a challenge that the exact mechanism of Sal B actions from the physiological constriction switches to pathological relaxation. The clinical aspects of heme oxygenase hinted its pharmacological actions in pathological status [13]. Further research on Sal B mechanisms might go on the way of systems biology [34].

Sal B for reducing PH might be used to explain the actions of medical plants in Chinese prescription for the ascitic patients with chronic hepatitis. It is an interesting clue to discover more effective candidates depending on the macrophage iNOS or HO-1 in portal triads, at least partly. Consequently, Sal B or its derivative might be exploited as a candidate to increase NO bioavailability or CO potency, especially from free radical damages in inflammatory diseases.
